# Model based analysis of real-time PCR data from DNA binding dye protocols

**DOI:** 10.1186/1471-2105-8-85

**Published:** 2007-03-09

**Authors:** Mariano J Alvarez, Guillermo J Vila-Ortiz, Mariano C Salibe, Osvaldo L Podhajcer, Fernando J Pitossi

**Affiliations:** 1 Gentron Research Unit, Arenales 1457 – 2° Piso, Buenos Aires C1061AAO, Argentina; 2 Gene Therapy Laboratory, Leloir Institute, CONICET, University of Buenos Aires, Patricias Argentinas 435, Buenos Aires C1405BWE, Argentina; 3 Neuroimmunomodulation and Gene Therapy Laboratory, Leloir Institute, CONICET, University of Buenos Aires, Patricias Argentinas 435, Buenos Aires C1405BWE, Argentina; 4 Joint Centers for Systems Biology, Columbia University, 1130 St Nicholas Avenue, New York, NY 10032, USA

## Abstract

**Background:**

Reverse transcription followed by real-time PCR is widely used for quantification of specific mRNA, and with the use of double-stranded DNA binding dyes it is becoming a standard for microarray data validation. Despite the kinetic information generated by real-time PCR, most popular analysis methods assume constant amplification efficiency among samples, introducing strong biases when amplification efficiencies are not the same.

**Results:**

We present here a new mathematical model based on the classic exponential description of the PCR, but modeling amplification efficiency as a sigmoidal function of the product yield. The model was validated with experimental results and used for the development of a new method for real-time PCR data analysis. This model based method for real-time PCR data analysis showed the best accuracy and precision compared with previous methods when used for quantification of *in-silico *generated and experimental real-time PCR results. Moreover, the method is suitable for the analyses of samples with similar or dissimilar amplification efficiency.

**Conclusion:**

The presented method showed the best accuracy and precision. Moreover, it does not depend on calibration curves, making it ideal for fully automated high-throughput applications.

## Background

The reverse transcription polymerase chain reaction (RT-PCR) is the most sensitive method for the detection of specific mRNAs [1]. However, due to the exponential nature of the PCR amplification process, small differences in amplification efficiency among samples may led to very different product yields, making RT-PCR unsuitable for quantitative purposes. The use of exogenously added standard sequences as internal competitors has overcome partially this issue [2-4], but the setting up of the system must be done for each target sequence taking into account multiple error sources [5], making competitive PCR unsuitable for high-throughput applications. The recent introduction of fluorescence techniques and instruments able to quantify the DNA content in each cycle had lead in the last years to the development of real-time PCR [6-8]. Because the high sensitivity of fluorescent product detection, real-time PCR does not rely on end-point analyses. Moreover, cycle-by-cycle data generated by real-time PCR provides information about the kinetics of the amplification process, overcoming the limitations of classical RT-PCR. Recently, the introduction of double-stranded DNA specific dyes [9] allowed the quantification of multiple targets without the need of specific fluorescent probes, making real-time PCR a popular method for microarray data validation [10].

Most currently used real-time PCR data analysis methods are based on determining the threshold cycle (*CT*), which is the cycle number at which a fixed amount of product is formed [11]. The comparative *CT *method, a broadly used semi-quantitative one, is based on the exponential description of the PCR process assuming constant amplification efficiency equal to 1 [12]. However, in our applications of SYBR-Green I real-time PCR, we have found amplification efficiency always lower that 1. Moreover, it has been shown that a difference as small as 4% in PCR efficiency could be translated to 400% error in comparative *CT *method based quantifications [13]. Thus, reliable quantitative real-time PCR depends on good estimations of PCR efficiency. Several methods have been proposed for amplification efficiency estimation. One of them uses a dilution curve to estimate the amplification efficiency of each target sequence [14], while the others estimate the amplification efficiency from single reaction data [13,15-17]. Here we introduce a new mathematical model based on the classic exponential description of the PCR, in which amplification efficiency was modelled as a sigmoid function of the product yield. The model was validated with experimental data and it was used for the development of a new method for real-time PCR data analysis. This model based method for real-time PCR data analysis (MoBPA) estimates PCR amplification efficiency from single sample reaction data, eliminating the need of calibration curves, which is a drawback for high-throughput implementations [18]. Moreover, MoBPA showed the highest accuracy and precision compared with previous methods when used for quantification of samples amplified with similar or dissimilar efficiency.

## Results and discussion

### The model

**Figure 1:**
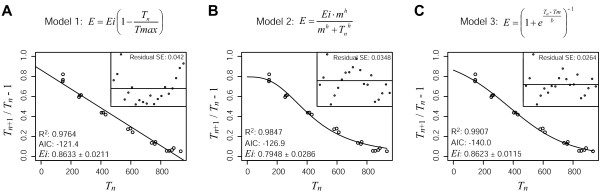
**Models for PCR amplification efficiency**. The effective amplification efficiency for each PCR cycle was calculated as *T*_*n*+1 _/*T*_*n *_– 1, where *T*_*n *_and *T*_*n*+1 _were the PCR product yield at cycles *n *and *n+*1 respectively. Data points are the effective amplification efficiency vs. PCR product yield from a representative PCR reaction performed in triplicate. Lines are the fit of models 1 (A), 2 (B) and 3 (C) to the experimental data. Inserts are the residuals for each fit. The determination coefficient (R^2^), corrected Akike's Information Criterion (AIC) and the best fit value for *Ei *± asymptotic standard error are shown in the graphs. *Ei *for model 3 was calculated from Eq. (3). Model 1 was fitted by linear regression, while models 2 and 3 were fitted by non-linear regression.

According to its discrete nature, the PCR process can be expressed by the difference equation,

*T*_*n*+1 _= *T*_*n*_·(1 +*E *_*n*_);     *E*_*n *_∈ (0,1)     (1)

where *T*_*n *_is the PCR product yield at cycle *n*, and *E *is the amplification efficiency of the reaction. In a previous work, we have modelled the amplification efficiency as a linear function of the product yield (model 1; Fig. 1A) [5]. However, a recent kinetic description of the real-time PCR showed a sigmoid relationship between the effective amplification efficiency and the product yield, either whether primer, nucleotides or DNA polymerase become limiting [19]. This prompted us to evaluate two additional empirical models describing the amplification efficiency as a function of PCR product yield: a three parameters, and a two parameters sigmoid models (models 2 and 3; Fig. 1B and 1C). Both models imply that the amplification efficiency change dynamically also during the exponential phase, which is in agreement with the work of Liu, et.al [15]. However, our models differ from the sigmoid description of the PCR reaction, because we model the amplification efficiency as a function of the product yield, instead of describing it as a function of the cycle number. The models were fitted to the same real-time PCR dataset comprising 1723 PCR reactions, with initial target amount spanning 9 orders of magnitude and CT values that range from 10 to 35.9. Despite the fact that the three models explained more than 95% of the data variance, only models 2 and 3 showed a random distribution of residuals (Fig. 1). To identify the best way of describing the amplification efficiency, we compared the models using a corrected form of Akaike's Information Criterion (AIC) [20]. Model 3 AIC value was lower than model 1 and 2 (p < 10^-15 ^and p < 10^-4^, Wilcoxon paired test, respectively). Thus, the following two parameters sigmoid expression for *E *was used through this work,

En=(1+e(Tn−Tmb))−1     (2)
 MathType@MTEF@5@5@+=feaafiart1ev1aaatCvAUfKttLearuWrP9MDH5MBPbIqV92AaeXatLxBI9gBaebbnrfifHhDYfgasaacH8akY=wiFfYdH8Gipec8Eeeu0xXdbba9frFj0=OqFfea0dXdd9vqai=hGuQ8kuc9pgc9s8qqaq=dirpe0xb9q8qiLsFr0=vr0=vr0dc8meaabaqaciaacaGaaeqabaqabeGadaaakeaacqWGfbqrdaWgaaWcbaGaemOBa4gabeaakiabg2da9maabmaabaGaeGymaeJaey4kaSIaemyzau2aaWbaaSqabeaadaqadaqaamaalaaabaGaemivaq1aaSbaaWqaaiabd6gaUbqabaWccqGHsislcqWGubavcqWGTbqBaeaacqWGIbGyaaaacaGLOaGaayzkaaaaaaGccaGLOaGaayzkaaWaaWbaaSqabeaacqGHsislcqaIXaqmaaGccaWLjaGaaCzcamaabmaabaGaeGOmaidacaGLOaGaayzkaaaaaa@444D@

where *Tm *and *b *are parameters to be fitted by non-linear regression. There is no relationship between these parameters and kinetic parameters such as *Fmax *and *k *[18], since our model is defined as a function of the product yield instead of the PCR cycle number.

The intrinsic amplification efficiency *Ei *(i.e. the putative amplification efficiency for a product (or template) amount equal zero)[5], is useful for *T*_0 _estimation (see below), and can be obtained from Eq. (2) as,

Ei=(1+e−(Tmb))−1     (3)
 MathType@MTEF@5@5@+=feaafiart1ev1aaatCvAUfKttLearuWrP9MDH5MBPbIqV92AaeXatLxBI9gBaebbnrfifHhDYfgasaacH8akY=wiFfYdH8Gipec8Eeeu0xXdbba9frFj0=OqFfea0dXdd9vqai=hGuQ8kuc9pgc9s8qqaq=dirpe0xb9q8qiLsFr0=vr0=vr0dc8meaabaqaciaacaGaaeqabaqabeGadaaakeaacqWGfbqrcqWGPbqAcqGH9aqpdaqadaqaaiabigdaXiabgUcaRiabdwgaLnaaCaaaleqabaGaeyOeI0YaaeWaaeaadaWcaaqaaiabdsfaujabd2gaTbqaaiabdkgaIbaaaiaawIcacaGLPaaaaaaakiaawIcacaGLPaaadaahaaWcbeqaaiabgkHiTiabigdaXaaakiaaxMaacaWLjaWaaeWaaeaacqaIZaWmaiaawIcacaGLPaaaaaa@4141@

### Model based estimation of the initial template amount (T0)

In dsDNA binding dye protocols, real-time thermocyclers generate fluorescence intensity data. For most applications in which the dsDNA binding dye is in great excess, it can be assumed that the fluorescence intensity is proportional to the amount of double stranded DNA (product yield) [18]. However, it has been shown that this is not always the case [21]. Our model parameters are fitted with fluorescence intensity data, thus the product yield (*T*_*n*_) will be expressed in arbitrary fluorescent units, as the initial template amount (*T*_0_). Since the fluorescent dye will be in great excess when compared to the initial template amount, we can assume that *T*_0 _is proportional to the initial template amount, and so the semiquantitative comparison between different samples using *T*_0 _is valid.

The simplest way to estimate *T*_0 _is assuming that amplification efficiency at cycle *CT *(*E*_*CT*_) is very similar to *Ei*. This assumption is usually correct because *CT *is defined at small amounts of PCR product. Then, solving Eq. (1) for *n *= *CT *and assuming constant amplification efficiency we obtain:

*T*_*CT *_= *T*_0 _(1 + *E*)^*CT *^    (4)

from which,

*T*_0 _= *T*_*CT *_(1 + *E*)^-*CT *^    (5)

*Ei *can be estimated from Eq. (3), and the efficiency parameters *b *and *Tm *can be obtained fitting Eq. (2) to real-time PCR data by non-linear regression (see methods for details). Then, *T*_0 _is calculated assuming *E *= *Ei*. Since Eq. (5) is a power function of the amplification efficiency, small errors in the estimation of *Ei *can be translated to strong errors in *T*_0 _estimation. These errors can be minimized using *CT *values as small as possibly, and estimating the amplification parameters with pooled data from replicates to improve *E*_*i *_estimation accuracy. Replicates must be done by splitting a master-mix containing all components of the PCR to minimize the variability introduced be the operator.

**Figure 2:**
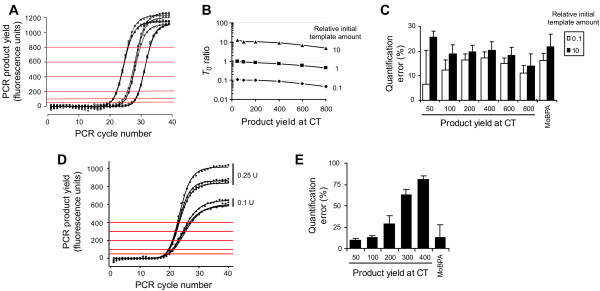
**Effect of CT on the initial template amount estimation**. (A) Product yield vs. cycle number for the amplification of three serial dilutions (0.1, 1 and 10) of cDNA from mouse midbrain with β-actin specific primers performed in triplicate. Horizontal lines show the values for the product yield at which *CT *was calculated. (B) Ratios between each *T*_0 _determination and *T*_0 _estimated for dilution 1 at the smallest *CT *value. *T*_0 _was calculated assuming constant amplification efficiency and using different amounts of PCR product for the estimation of *CT *values. Data points are the mean for triplicates. (C) Relative error for the quantification of data presented in A. Bars are the relative error of quantification as percentage (mean ± SEM for triplicates). (D) Real-time PCR amplification of the same mouse midbrain cDNA sample with β_2 _microglobulin specific primers using 0.1 and 0.25 units of Taq DNA polymerase. Horizontal lines show the values for the product yield at which *CT *was calculated. (E) Relative error for the quantification of data presented in D. Bars are the relative error of quantification as percentage (mean ± SEM for triplicates).

To test this method, we amplified three serial dilutions of cDNA from mouse midbrain with β-actin specific primers. Pooled data from triplicate experiments were used for *Ei *estimation, which were 0.835, 0.852 and 0.856 for dilutions 10, 1 and 0.1, respectively. Estimations of *T*_0 _from this data were accurate and precise for *CT *values calculated from small PCR product yields, but led to different absolute quantification results for higher *CT *values (Fig. 2A and 2B). However, since amplification efficiencies are similar, the relative error of quantifications was independent of the product yield at which the *CT *was calculated (Fig. 2C). To test the performance of the *CT *method when amplification efficiencies are not the same, we amplified the same amount of mouse midbrain cDNA with β_2_-microglobulin specific primers, but using different amounts of Taq DNA polymerase (Fig. 2D). Amplification efficiencies estimated with our approach from triplicate results were 0.855 and 0.915 for 0.1 and 0.25 units of Taq DNA polymerase, respectively. Then, an increase of only 7% in amplification efficiency led to errors in the quantifications that were directly related to the PCR product yield at which the CT was calculated (Fig. 2E).

We formulated an alternative more robust method for *T*_0 _estimation that does not rely on product threshold determinations. This alternative method is based on the fit of Eq. (1) and (2) to real-time PCR data by non-linear regression to obtain the best-fit estimators for the parameters *b*, *Tm *and *T*_0_. This approach does not assume constant amplification efficiency and estimates *T*_0 _from all the data points that fall into the exponential-linear growth phase instead of using only the product yield at cycle *CT*. Analysis of experimental results showed that *T*_0 _estimations were precise and accurate (Table 1). However, fitting the model to experimental data showed a strong correlation between *T*_0 _and both model 3 parameters *b *and *Tm*. This means that experimental data do not define the model unambiguously, leading to large uncertainty in the estimation of *T*_0 _(see standard error of *T*_0 _in Table 1). To overcome this problem, we propose a two-step procedure called Model-Based method for real-time PCR data Analysis (MoBPA). In the first step of the MoBPA procedure, we obtain good estimators for *b *and *Tm *parameters by fitting model 3 (Eq. 2) to experimental data. In the second step, we replace *b *and *Tm *in Eq. 2 with the values estimated in step 1, and then fit Eq. 1 to experimental data to obtain a good estimator for *T*_0_. *T*_0 _estimations by MoBPA were as precise and accurate as using the CT method for small PCR product yield-derived CT values (Fig. 2C and 2E), and the asymptotic standard errors were approximately 10 fold smaller (Table 1).

**Table 1:** Estimation of model parameters.

	**A**					**B**		
		
			Mean ±	Correlation				Mean ±
Dilution	*T*_0_	SE	SEM	*T*_0 _× *b*	*T*_0 _× *Tm*	*T*_0_	SE	SEM
10	8.52	2.27	8.63 ±	0.963	-0.97	12.4	0.29	12.17 ±
10	7	2	0.98	0.963	-0.977	11.2	0.34	0.52
10	10.4	1.95		0.963	-0.969	12.9	0.36	

1	0.42	0.06		0.967	-0.962	0.981	0.018	
1	1.08	0.56	1 ± 0.32	0.964	-0.948	0.804	0.024	1 ± 0.12
1	1.51	0.74		0.965	-0.977	1.21	0.043	

0.1	0.09	0.04	0.073 ±	0.968	-0.965	0.121	0.0032	0.12 ±
0.1	0.065	0.015	0.0087	0.969	-0.974	0.111	0.0026	0.0028
0.1	0.063	0.023		0.968	-0.921	0.117	0.0028	

Estimation of *T*_0_, *b *and *Tm *by fitting Eq. (1) and (2) to experimental data (A), and estimation of *T*_0 _by fitting Eq. (1) and (2) to experimental data using *b *and *Tm *values previously obtained from the fit of Eq. (2) to experimental amplification efficiencies (B). Shown is the dilution of mouse midbrain cDNA used for each real-time PCR run, the best fit values for *T*_0_, the asymptotic estimation of the standard error (SE), and the *T*_0 _mean value ± standard error of the mean (SEM). For (A), the correlation between *T*_0 _and the other parameters estimated by non-linear regression is also shown.

### Comparison of MoBPA with previous methods

Next, we evaluated the performance of different methods for quantification when amplification efficiency for different samples is not the same. For this, we analysed two different datasets: 1) *in-silico *generated data of real-time PCR runs starting at the same initial template amount but differing in their intrinsic amplification efficiency; and 2) real experimental data obtained by amplifying the same amount of mouse midbrain cDNA with β_2_-microglobulin specific primers, but with different amounts of Taq DNA polymerase (Fig. 2D).

Simulation data was generated *in-silico *from eqs. (1) and (2), using parameters that resemble real PCR runs. Results from amplification of mouse midbrain cDNA with β-actin and β_2_-microglobulin specific primers were used to estimate plausible values for the simulation parameters. The analysis of these simulation results must be taken with care, because we used the same model for generating and fitting the data, so some overfitting is expected. However, we also evaluated the performance of the different methods on real PCR data. To obtain different amplification efficiency in our PCR runs, we used two different amount of DNA polymerase. Efficiencies estimated with our approach from triplicate results were 0.855 and 0.915 for 0.1 and 0.25 units of Taq DNA polymerase, respectively. The use of more than 0.25 units of DNA polymerase did not led to additional increments in amplification efficiency, while PCR with less that 0.1 units of DNA polymerase showed no product. We also tried to partially reduce the PCR amplification efficiency by adding Mg^2+ ^chelating agents like EDTA, by increasing the amount of dNTPs, by lowering the amount of Mg^2+^, and by adding known DNA polymerase inhibitors like phenol. However, we could not find the appropriate conditions to achieve the partial inhibition of DNA polymerase in a reproducible way.

Both *in-silico *and experimental real-time PCR data were analysed with different methods. The simpler and widespread used methods are based on *CT *value determinations, assuming constant amplification efficiency during the exponential phase of the PCR and among different samples [11,22]. Then, from Eq. (5) is easy to solve the initial template amount ratio between two samples as:

T0AT0B=(1+E)CTB−CTA     (6)
 MathType@MTEF@5@5@+=feaafiart1ev1aaatCvAUfKttLearuWrP9MDH5MBPbIqV92AaeXatLxBI9gBaebbnrfifHhDYfgasaacH8akY=wiFfYdH8Gipec8Eeeu0xXdbba9frFj0=OqFfea0dXdd9vqai=hGuQ8kuc9pgc9s8qqaq=dirpe0xb9q8qiLsFr0=vr0=vr0dc8meaabaqaciaacaGaaeqabaqabeGadaaakeaadaWcaaqaaiabdsfaunaaBaaaleaacqaIWaamcqWGbbqqaeqaaaGcbaGaemivaq1aaSbaaSqaaiabicdaWiabdkeacbqabaaaaOGaeyypa0ZaaeWaaeaacqaIXaqmcqGHRaWkcqWGfbqraiaawIcacaGLPaaadaahaaWcbeqaaiabdoeadjabdsfaunaaBaaameaacqWGcbGqaeqaaSGaeyOeI0Iaem4qamKaemivaq1aaSbaaWqaaiabdgeabbqabaaaaOGaaCzcaiaaxMaadaqadaqaaiabiAda2aGaayjkaiaawMcaaaaa@44DA@

where *T*_0 _is the initial template amount, *E *is the amplification efficiency, and *CT*_*i *_the threshold cycle for each sample. The simplest form of threshold-based methods even assumes amplification efficiency equal 1. Thus, the ratio of initial template amounts between two samples will be 2^Δ*CT *^[12]. Because of the exponential nature of this expression, amplification efficiencies below 1 will lead to unreliable quantifications. Amplification efficiency below 1 is frequent and indeed, most of our real-time PCR reactions showed amplification efficiencies between 0.7 and 0.95. To overcome this limitation, a dilution series based method for amplification efficiency estimation has been developed [14,23]. A curve is constructed by amplification of serial dilutions of one reference sample and plotting the resulting *CT *values against the base 10 logarithm of the dilution factor. Assuming constant amplification efficiency between dilutions and over the number of thermocycles required to reach *CT*, the amplification efficiency can be obtained from Eq. (4) as *E *= 10^-1/slope^-1. However, sample contamination with salt, phenol, chloroform, etc. may result in a lower-than-expected PCR efficiency [13,24,25]. Dilution of this sample will also dilute the contaminant decreasing its effect on the PCR reaction, thereby increasing the PCR efficiency with each dilution step. Indeed, we usually observe such dilution effect on PCR amplification efficiency, see for example data presented in Fig. 2A, in which estimated efficiency from the dilution series was 0.853, while estimations of the intrinsic amplification were 0.835, 0.852 and 0.856 for dilutions 100, 10 and 1, respectively. Amplification efficiency estimations from such dilution series will be inaccurate, introducing a bias in the quantification. Moreover, contaminants can quantitatively and qualitatively differ between samples, thus, the assumption of equal amplification efficiencies between samples can lead to strong errors in quantifications.

**Figure 3:**
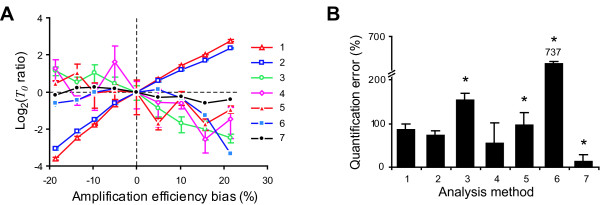
**Effect of amplification efficiency over the quantifications performed by different methods**. (A) *In-silico *generated PCR data with initial template amount *T*_0 _= 0.001 and different intrinsic amplification efficiencies (*Ei*) ranging from 0.65 to 0.972 analysed by different methods (see below). Data points represent the base 2 logarithm of the ratio between *T*_0 _estimations from each simulated reaction and efficiency 0.8 ones vs. the amplification efficiency bias as mean ± SEM of triplicates. (B) Analysis of experimental results by different methods (see below). Bars represent the error of quantifications as mean ± SEM for triplicates. Bars marked with (*) are under-estimations, conversely, the rest of the bars are over-estimations. Method 6 under-estimated *T*_0 _by 737%, note that it is out of scale in the graph. Data was analysed with the *CT *method assuming constant amplification efficiency equal to 1 {1}; with the *CT *method assuming constant amplification efficiency equal to 0.8 for *in-silico *data, or 0.855 for experimental data {2}; assuming constant amplification efficiency that was estimated from two threshold values {3} [15]; using the assumption-free analysis proposed by Ramakers et.al. {4} [13]; using the standardized determination of PCR efficiency from single reaction proposed by Tichopad et.al. {5} [16]; using the sigmoid model proposed by Liu et. al. {6} [17]; and with our model based real-time PCR analysis method (MoBPA) {7}.

Analysis of *in-silico *PCR results showed that the bias in quantifications by these methods is directly related to the difference in amplification efficiency between templates. In such a way, a 20% difference in amplification efficiency leaded to 11 and 7.4 fold bias in quantifications depending on whether efficiency 1 or reference sample efficiency (0.8) was used for calculations (Fig. 3A). Accordingly, the quantification of β2-microglobulin was 86% and 74% overestimated by the use of efficiency 1 or 0.855 in threshold-based methods, respectively (Fig. 3B). Despite the poor accuracy of these methods, *T*_0 _estimation from replicated samples was very precise (mean CV: 6%, range: 0.75–12%), possibly because these approaches are only affected by errors in *CT *estimation. Similar precision was obtained with experimental real-time PCR results (Fig. 3B), and it was previously reported by other authors [26].

Four different methods for single reaction amplification efficiency estimation have been proposed. All of them use the kinetic data generated by real-time PCR cyclers, they do not assume equivalent amplification efficiency among samples and have the additional advantage that no dilutions curve is needed. Three of them assume constant amplification efficiency during the exponential phase of the PCR and estimate it from the few data points that fall into this phase [13,15,16]. The simplest one is based on the product yield measured at two thresholds along the exponential phase of the PCR, from which the amplification efficiency is estimated as *E *= (*T*_1 _/*T*_2_)^1/(*CT*1-*CT*2) ^- 1 [15]. Then, the initial template amount is calculated from Eq. (5). Based on the same model for the PCR reaction, Ramakers et. al. proposed the use of all the points of the background subtracted and logarithm transformed PCR data that fall into a "window-of-linearity", for amplification efficiency estimation by linear regression [13]. Recently, Tichopad et.al. introduced a similar approach based on a new statistical method for the identification of the exponential phase and estimation of amplification efficiency by non-linear regression [16]. Analysis of *in-silico *generated results with different amplification efficiencies showed better accuracy of these three approaches when compared with the threshold-based methods, since they introduced only 2.3 – 4.8 fold bias in quantifications (Fig. 3A). However, since *T*_0 _determinations depend on both efficiency and *CT *estimations, precision in quantification of replicates was very poor (mean CV: 58%, range: 19–134%). Indeed, quantification of samples that differ less than 10% in amplification efficiency were more accurate when analysed with the Pfaffl or efficiency 1 threshold-based methods [12,14], most likely due to the poor precision of single reaction based efficiency estimations. These observations were further confirmed with experimental real-time PCR data (Fig. 3B).

Recently, Liu et.al. proposed a three parameters sigmoidal function for modelling the whole kinetic process of real-time PCR. Then, the initial template amount is estimated after fitting the sigmoidal model to background subtracted real-time PCR results [17]. Analysis of *in-silico *PCR results showed a good accuracy and moderate precision of this method for most PCR simulations, with a fold bias < 1.4 and a mean CV of 12% (range: 5–21%) (Fig. 3A). However, the model presented systematic deviations from the data that were particularly noted with amplification efficiencies above 0.9, both with experimental and *in-silico *generated data. These deviations had a strong effect on *T*_0 _estimation, leading to unreliable quantifications (Fig. 3B). Similar deviations were recently described in the plateau phase by Rutledge, who showed that elimination of these data points improve the accuracy of this method [18].

Finally, quantification from the same *in-silico *and experimental real-time PCR results with our model based approach showed the highest accuracy and precision. Analysis of *in-silico *data showed that 20% difference in amplification efficiency between samples only led to 0.31 fold bias, with a mean CV of only 2.6% (range: 0.8–4.2%) (Fig. 3A). Moreover, an increase of 7% in experimental real-time PCR amplification efficiency only led to 13% under-estimated quantification (mean CV: 10.5%) (Fig. 3B).

To test the reliability of the different methods in conditions of similar amplification efficiency between samples, we amplified three dilutions of mouse midbrain cDNA with β-actin specific primers. Among threshold-based methods, the use of amplification efficiency estimated for each experiment from the dilution series [14] produced the best results (Table 2). Most methods for the estimation of amplification efficiency from single reaction results introduced strong deviations in the quantification. Only the approach suggested by Tichopad et.al. [16] showed moderate deviations (Table 2). Similar observation have been reported by Peirson et.al., who showed that estimation of amplification efficiency from single reactions data introduces systemic errors and increases the assay noise [27], suggesting that these methods should be used only when the amplification efficiency between samples is not the same. A statistical method for the detection of samples with dissimilar efficiencies, called kinetic outlier detection (KOD), was recently developed [28]. KOD method can be used to decide whether to use single reaction or global amplification efficiency estimated from dilution series for quantifications. To note, our model based approach gave the most accurate and precise results, which were similar to the ones produced by Pfaffl approach [14] (Table 2), indicating that MoBPA produce reliable quantifications no matter whether samples are amplified with similar or different efficiency.

**Table 2:** Analysis of real-time PCR results with similar amplification efficiency among samples. Data represent the quantification of dilutions 0.1 and 10 as the mean ± SEM for 12 experiments performed in triplicate.

**Dilution**	**Analysis method**						
	1	2	3	4	5	6	7
0.1	0.084 ± 0.003	0.11 ± 0.0033	0.57 ± 0.30	0.37 ± 0.19	0.75 ± 0.45	0.71 ± 0.63	0.12 ± 0.022
1	1 ± 0.019	1 ± 0.017	1 ± 0.11	1 ± 0.20	1 ± 0.12	1 ± 0.10	1 ± 0.018
10	14 ± 0.680	10 ± 0.35	242 ± 206	83 ± 52	15 ± 5.5	48 ± 1.0	10 ± 1.1

(1)*CT *method assuming constant amplification efficiency equal to 1 [12].

(2) *CT *method with amplification efficiency estimated from a dilution series [14].

(3) Amplification efficiency estimated at two product yield thresholds [15].

(4) Amplification efficiency estimated with LinRegPCR software [13].

(5) Amplification efficiency estimated with the Tichopad et.al. approach [16].

(6) Amplification efficiency estimated from the model proposed by Liu et.al. [17].

(7) Our model based real-time PCR analysis method (MoBPA).

Our model assumes that the signal is proportional to the amount of product, which is often the case for SYBR-Green I real-time PCR performed with saturating concentrations of dye. In such conditions centrally symmetric amplification curves are expected. However, in TaqMan applications, where the Taq DNA polymerase digests a probe labelled with a fluorescent reporter and quencher dye, the signal diverges from the product resulting in non-symmetric amplification curves (Supplementary Fig. 1A in Additional file 1) [8]. To test whether our approach is also suitable for TaqMan data analysis, we quantified β-actin and hypoxanthine phosphoribosyl transferase (HPRT) in 1/10 dilutions of the same cDNA sample using TaqMan probes. The error of quantifying a 10-fold concentrated sample was only slightly higher using TaqMan when compared to SYBR-Green I (Fig. 2C and Supplementary Fig. 1B in Additional file 1). These results suggest that MoBPA is robust enough to deal with non-symmetric amplification curves, possibly because it makes use of only the data points that fall into the exponential-lineal growth phases of the PCR.

## Conclusion

Following the emergence of functional genomic methodologies, the development of high-throughput methods for microarray-derived data validation is becoming indispensably. Nevertheless, high-throughput quantification by real-time PCR is difficult to achieve, primarily due to deficiencies of the threshold-based methodologies, which require reliable estimation of amplification efficiencies [27]. In addition to the technical challenge of generating dilution curves for each target sequence, these methods assume similar amplification efficiencies between samples, and this assumption has been reported to be invalid [13,24,25]. Here we introduce a new method for the analysis of real-time PCR results termed: Model Based method for real-time PCR data Analysis (MoBPA). The new method produced the most accurate and precise quantifications when compared to previous methods regardless of whether samples were amplified with similar or different amplification efficiencies. Moreover, no calibration curve is needed for quantifications, since amplification efficiency is estimated directly from real-time PCR data. MoBPA provides many of the fundamental capabilities required for fully automated high-throughput quantification [18], including routine assessment of amplification efficiency within individual samples and no need of PCR-generated standard curves. In addition, quantitative data can be easily derived from arbitrary fluorescence units, simply applying a calibration factor that relates fluorescence to DNA mass [18]. In conclusion, MoBPA combines the well accepted exponential model of the PCR reaction with a sigmoid description of amplification efficiency to obtain a powerful method for real-time PCR data analysis that expand the applicability of real-time PCR to fully automated high-throughput applications.

## Methods

### RNA extraction and reverse transcription

Mice were killed by cervical dislocation and brains removed. Midbrains were dissected, snap frozen in liquid nitrogen and stored at -80°C until RNA extraction. Total RNA was isolated using TRIzol Reagent (Invitrogen, MD), genomic DNA contaminant was removed using DNAse I (Ambion, Inc., TX), and mRNA was purified by MicroPoly(A)Pure kit (Ambion, Inc., TX). First-strand complementary DNA was synthesized at 42°C by priming with oligo-dT_12–18 _(Invitrogen, MD) and using SuperScriptII reverse transcriptase according to the protocol provided by the manufacturer (Invitrogen, MD).

### Polymerase chain reaction

PCR amplifications were obtained using an Icycler IQ Real-Time PCR Detection System (BioRad, CA). cDNA samples were assayed by triplicate. PCR reactions were performed in a final volume of 25 μl containing 1 μl of cDNA, 2.5 μl of the reaction buffer (200 mM Tris-HCl pH 8.4, 500 mM KCl), 3 mM MgCl_2_, 0.3 mM of dNTPs mix, 0.2 nM of each primer, 0.3 × SYBR-Green I (Molecular Probes, OR), 100 μg/ml BSA, 0.25 μl ROX Reference Dye (Invitrogen, MD), 1% glycerol, and 1.25 U of Taq Platinum Polymerase (Invitrogen, MD). The primer sequences used were: β2-microglobulin, sense: TGA CCG GCT TGT ATG CTA TC and antisense: CAG TGT GAG CCA GGA TAT AG; β-actin, sense: CAA TGT GGC TGA GGA CTT TG and antisense: ACA GAA GCA ATG CTG TCA CC. PCR was performed as follows: one initial cycle of 94°C for 2.5 min, 40 cycles of 94°C for 30 sec, 58°C for 30 sec, and 72°C for 15 sec. TaqMan real-time PCRs were performed as the SYBR-Green I assays, but with no addition of SyBrGreen I and with the following primers and probes: β-actin sense: AGA AAA TCT GGC ACC ACA CC, antisense: CAG AGG CGT ACA GGG ATA GC, and probe: ACC GCG AGA AGA TGA CCC AGA TCA T; HPRT sense: AGA CTG AAG AGC TAT TGT AAT, antisense: CAG CAA GCT TGC GAC CTT GAC, and probe: TGC TTT CCT TGG TCA GGC AGT ATA.

### Analysis of real-time PCR data

ROX base-line corrected real-time PCR results were analysed with different methods as described [12-17] using the R-System v2.2.0 [29] or the LinRegPCR software [13]. For the LinRegPCR software, we used the default fit option, which iteratively searches for lines consisting in 4 to 6 data points with the highest R^2 ^value. Then we inspected the fit for each PCR curve and manually corrected the windows of linearity when needed.

The implementation of our method comprise the following steps: 1) Identification of ground, exponential, lineal growth and plateau phases, 2) background subtraction, 3) effective amplification efficiency estimation and fitting eq (2) to experimental data, 4) initial template amount calculation.

The ground phase was identified as described [16], using a p-value cut-off of 0.01, while the beginning and end of the lineal growth phase was identified as the second derivative maximum and minimum, respectively, of a four parameters sigmoid function (eq. 7) fitted to the data (Supplementary Figure 2A in Additional file 1).

y=(mi−ma)chch+xh     (7)
 MathType@MTEF@5@5@+=feaafiart1ev1aaatCvAUfKttLearuWrP9MDH5MBPbIqV92AaeXatLxBI9gBaebbnrfifHhDYfgasaacH8akY=wiFfYdH8Gipec8Eeeu0xXdbba9frFj0=OqFfea0dXdd9vqai=hGuQ8kuc9pgc9s8qqaq=dirpe0xb9q8qiLsFr0=vr0=vr0dc8meaabaqaciaacaGaaeqabaqabeGadaaakeaacqWG5bqEcqGH9aqpdaWcaaqaamaabmaabaGaemyBa0MaemyAaKMaeyOeI0IaemyBa0MaemyyaegacaGLOaGaayzkaaGaem4yam2aaWbaaSqabeaacqWGObaAaaaakeaacqWGJbWydaahaaWcbeqaaiabdIgaObaakiabgUcaRiabdIha4naaCaaaleqabaGaemiAaGgaaaaakiaaxMaacaWLjaWaaeWaaeaacqaI3aWnaiaawIcacaGLPaaaaaa@4491@

Here, *y *is the PCR product yield (fluorescence units), *x *is the cycle number, *mi *and *ma *are the signal-offset and saturation value, respectively, *c *is the point of inflexion and *h *is the exponent parameter. Non-linear least square regressions were performed with a Gauss-Newton algorithm implemented in the nls function of the R-system software.

The background level was calculated as the last ground phase data point, which in tern was estimated by a linear regression over the last five data points of this phase [16].

The effective amplification efficiency for each PCR cycle (*E*_*n*_) was solved from Eq. (1) as *E*_*n *_= *T*_*n*+1 _/*T*_*n *_- 1 [5] and calculated using the background subtracted data. Then, the parameters *b *and *Tm *were estimated by fitting Eq (2) to the experimental effective amplification efficiency by non-linear regression. We only used data points that fall into the exponential and linear growth phase of the amplification curve, because efficiency calculated at the early ground phase and late plateau phase showed strong dispersion due to experimental background noise or showed no information, respectively (Supplementary Figure 2 in Additional file 1). Finally, the initial template amount *T*_0 _was estimated by fitting our discrete model, shown below in R-code:

T <- function(x,t0,b,Tm) {

   output <- NULL

   for (i in 1:length(x)) {

      t00 <- t0

      for (ii in 1:x [i]) t00 <- t00*(1+1/(1+exp((t00-Tm)/b)))

      output <- c(output,t00)

   }

   return(output)

}

to background subtracted experimental data by the nls function of R-system, using the values for the parameters *b *and *Tm *estimated previously.

Our method was implemented in the R-System. The source code and Windows binary of MoBPA package are available for non-commercial research use (see Additional files 2 and 3).

### Quantification error and Akaike's Information Criterion

For calculating the quantification error, we defined a reference sample (*RS*) and a target sample (*TS*), which are derived from the same original sample and differ by a known dilution factor or PCR amplification efficiency. We calculated the relative bias as *RE *= (*TS *- *RS*)/*RS*, and the quantification error as *SQE *= RE * 100 for *RE *≥ 0, or *SQE *= -100 * *RE */(1 + *RE*) for RE < 0.

For comparing alternative models we used a corrected form of the Akaike's Information Criterion (*AIC*), defined as:

AIC=Nlog⁡(SSN)+2K+2K(K+1)N−K−1     (7)
 MathType@MTEF@5@5@+=feaafiart1ev1aaatCvAUfKttLearuWrP9MDH5MBPbIqV92AaeXatLxBI9gBaebbnrfifHhDYfgasaacH8akY=wiFfYdH8Gipec8Eeeu0xXdbba9frFj0=OqFfea0dXdd9vqai=hGuQ8kuc9pgc9s8qqaq=dirpe0xb9q8qiLsFr0=vr0=vr0dc8meaabaqaciaacaGaaeqabaqabeGadaaakeaacqWGbbqqcqWGjbqscqWGdbWqcqGH9aqpcqWGobGtcyGGSbaBcqGGVbWBcqGGNbWzdaqadaqaamaalaaabaGaem4uamLaem4uamfabaGaemOta4eaaaGaayjkaiaawMcaaiabgUcaRiabikdaYiabdUealjabgUcaRmaalaaabaGaeGOmaiJaem4saS0aaeWaaeaacqWGlbWscqGHRaWkcqaIXaqmaiaawIcacaGLPaaaaeaacqWGobGtcqGHsislcqWGlbWscqGHsislcqaIXaqmaaGaaCzcaiaaxMaadaqadaqaaiabiEda3aGaayjkaiaawMcaaaaa@4E8D@

where *N *is the number of data points, *K *is the number of parameters fitted by the regression plus one, and *SS *is the sum of the square of the vertical distances of the points from the curve [20].

### *In silico *generation of PCR data

Simulation data was generated *in silico *from eqs. (1) and (2), using parameters that resemble real PCR runs. Results from amplification of mouse midbrain cDNA with β-actin and β_2_-microglobulin specific primers were used to estimate plausible values for the simulation parameters. In such a way, the initial template amount in fluorescent arbitrary units (*T*_0_) was set to 10^-3^, and the product amount at the PCR plateau phase (*Tmax*) was set to 1200. The amplification parameters *b *and *Tm *were solve from eq. (2) and (3) as,

b=Tnlog⁡En−EiEnEi−EiEn,Tm=blog⁡(1Ei−1),
 MathType@MTEF@5@5@+=feaafiart1ev1aaatCvAUfKttLearuWrP9MDH5MBPbIqV92AaeXatLxBI9gBaebbnrfifHhDYfgasaacH8akY=wiFfYdH8Gipec8Eeeu0xXdbba9frFj0=OqFfea0dXdd9vqai=hGuQ8kuc9pgc9s8qqaq=dirpe0xb9q8qiLsFr0=vr0=vr0dc8meaabaqaciaacaGaaeqabaqabeGadaaakeaacqWGIbGycqGH9aqpdaWcaaqaaiabdsfaunaaBaaaleaacqWGUbGBaeqaaaGcbaGagiiBaWMaei4Ba8Maei4zaC2aaSaaaeaacqWGfbqrdaWgaaWcbaGaemOBa4gabeaakiabgkHiTiabdweafnaaBaaaleaacqWGPbqAaeqaaOGaemyrau0aaSbaaSqaaiabd6gaUbqabaaakeaacqWGfbqrdaWgaaWcbaGaemyAaKgabeaakiabgkHiTiabdweafnaaBaaaleaacqWGPbqAaeqaaOGaemyrau0aaSbaaSqaaiabd6gaUbqabaaaaaaakiabcYcaSiabdsfaujabd2gaTjabg2da9iabdkgaIjGbcYgaSjabc+gaVjabcEgaNnaabmaabaWaaSaaaeaacqaIXaqmaeaacqWGfbqrdaWgaaWcbaGaemyAaKgabeaaaaGccqGHsislcqaIXaqmaiaawIcacaGLPaaacqGGSaalaaa@59A8@

Then, for *T*_*n *_= *Tmax*, *E*_*n *_tends to zero, so we calculated approximated values for *b *and *Tm *using *T*_*n *_= *Tmax *= 1200 and *E*_*n *_= 0.001.

Zero mean, normally distributed random noise was added to the *in-silico *results. The background mean (*Bg*) and intra- and inter-replicates standard deviation (*intraSD *and *interSD*) were estimated from 48 triplicate samples to be 625, 17.94 and 19.06 respectively. The mean standard deviation for fluorescence intensity in the early ground phase of each PCR reaction (*mSD*), also estimated from experimental data, was 4.9. We found no correlation between the standard deviation of fluorescence intensity in the early ground phase of each PCR reaction and *Bg *values, thus, random noise generated from a normal distribution with mean 0 and standard deviation *mSD *was added to *in-silico *PCR data regardless of the background fluorescence level.

## Authors' contributions

MJA carried out the design of the study, participated in data analysis and drafted the manuscript. GJV carried out the real time PCR. MCS participated in data collection and analysis. OLP and FJP participated in the design of the study and critically revised the manuscript. All authors read and approved the final manuscript.

## Supplementary Material

Additional File 1 supplementary figures 1 and 2. Click here for file

Additional File 2 Source files for the R-system package MoBPA. Click here for file

Additional File 3 Windows binary files for the R-system package MoBPA. Click here for file
