# Supplementary material for: Model based analysis of real-time PCR data from DNA binding dye protocols
Source: BMC Bioinformatics. 2007 Mar 9;8:85. doi: 10.1186/1471-2105-8-85 (PMC1838433; doi:10.1186/1471-2105-8-85)
Supplement: Additional File 1 — supplementary figures 1 and 2. [file 1471-2105-8-85-S1.pdf]

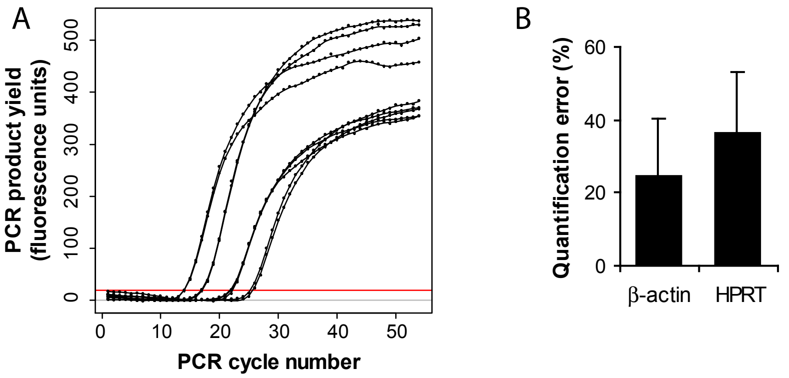


**Supplementary figure 1 - Analysis of TaqMan data using MoBPA**

(A) Product yield vs. cycle number for the amplification of two serial dilutions (1x and 10x) of the same cDNA sample with β-actin and HPRT specific primers using TaqMan. (B) Relative error for the quantification of data presented in A using MoBPA. Bars are the relative error of quantification as percentage (mean +/- SEM for duplicates).

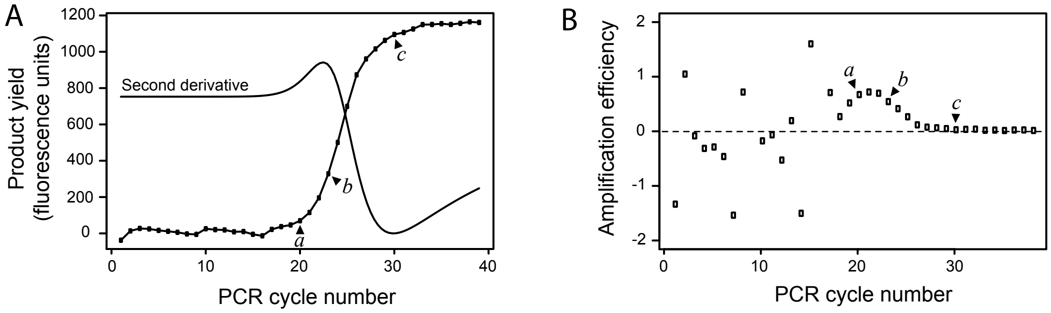


**Supplementary figure 2 - Identification of the different phases of the amplification curve**

(A) Representative experimental real-time PCR curve, it's second derivative, the first and last points of the exponential phase (a and b), and the last point of the linear growth phase (c). The second derivative of the PCR curve was calculated from the fit of Eq. 7 (see methods section) to experimental data.

(B) Experimental amplification efficiency for the same real-time PCR run as in (A). Note that dispersion due to background noise is strong at the early ground phase.
