# Supplementary material for: Model based analysis of real-time PCR data from DNA binding dye protocols
Source: BMC Bioinformatics. 2007 Mar 9;8:85. doi: 10.1186/1471-2105-8-85 (PMC1838433; doi:10.1186/1471-2105-8-85)
Supplement: Additional File 3 — Windows binary files for the R-system package MoBPA. [file 1471-2105-8-85-S3.zip › MoBPA.zip!/MoBPA/html/00Index.html]

R: Real Time PCR analysis tools

# Real Time PCR analysis tools

---

## Documentation for package `MoBPA' version 1.0

## Help Pages

|  |  |
| --- | --- |
| qpcr | Analysis of real-time amplification curves |
