# Supplementary material for: Model based analysis of real-time PCR data from DNA binding dye protocols
Source: BMC Bioinformatics. 2007 Mar 9;8:85. doi: 10.1186/1471-2105-8-85 (PMC1838433; doi:10.1186/1471-2105-8-85)
Supplement: Additional File 3 — Windows binary files for the R-system package MoBPA. [file 1471-2105-8-85-S3.zip › MoBPA.zip!/MoBPA/html/mobpa.html]

R: Analysis of real-time amplification curves

|  |  |
| --- | --- |
| qpcr {MoBPA} | R Documentation |

## Analysis of real-time amplification curves

### Description

This function analyzes real-time PCR reactions

### Usage

```
qpcr(input, output = "", replic = 3, bg = 0.01, autoct = T, gp = 1, lp = 1,
p.outlier = 1e-08, useEi = T, gtype = "pdf", graphics = F)
```

### Arguments

|  |  |
| --- | --- |
| `input` | Either a pcr.data matrix or a character string indicating the name of the input file. The input file must be a plain tab-delimited text file containing the baseline-corrected fluorescence values. Each column is a diferent sample and each row represent a PCR cycle. The first two rows are for annotations: the first one contains a unique identifier for each sample, and the second row contains the factors for identification of technical replicates |
| `output` | Optional character string indicating the name of the output file for graphics |
| `replic` | Only used if `input` is a matrix. Either a number indicating the amount of technical replicates, or a vector of sample labels |
| `bg` | p-value for the externally studentized residuals algorithm used for background phase identification |
| `autoct` | Logical, whether CT values should be calculated automatically |
| `gp` | Integer indicating the number of cycles to be added at the end of the ground phase for calculating the start of the exponential phase |
| `lp` | Integer indicating the number of cycles to be substracted from the start of the plateau phase for calculating the end of the exponential phase. we recommend to use 1 for CYBR-green applications and -3 for Taqman applications |
| `p.outlier` | Number between 0 and 1 indicating the p-value for residuals outlier detection during Ei estimation |
| `useEi` | Logical, whether pcr efficiency parameters should be fixed during initial template amount estimation by nonlinear regression |
| `gtype` | Character string indicating the type of the graphics file the function will produce. They can be: jpeg  jpeg file type, which is a bitmap file type  pdf  pdf file type, which is a vector file type  emf  enhanced windows metafile file type, which is a vector file type |
| `graphics` | Logical, whether graphics should be produced |

### Value

A data frame with the following components:

|  |  |
| --- | --- |
| `Reaction` | Reaction identification |
| `Ct` | Cycle threshold CT |
| `Efi` | Estimated intrinsic amplification efficiency |
| `To` | Estimated initial template amount |
| `Eti_NLR` | Intrinsic amplification efficiency estimated for individual reactions by non-linear regression |
| `To_NLR` | Initial template amount estimated by non-linear regression |

### Author(s)

Mariano Javier Alvarez malvarez@c2b2.columbia.edu

### Examples

```
#pcr.txt file contains data for 3 serial dilutions of mouse brain cDNA
pkgpath <- .find.package("MoBPA")
qpcr(input=file.path(pkgpath,"data","pcr.txt"), graphics=T)
```

---

[Package *MoBPA* version 1.0 Index]
